# The Foreign Journals: Anatomy and Physiology

**Published:** 1838-10

**Authors:** 


### PART THIRD.

### Selections from the British and Foreign Journals.

### I. THE FOREIGN JOURNALS.

### ANATOMY AND PHYSIOLOGY.

*Experiments* ON THE INTRODUCTION OF AIR INTO THE VEINS, *made by* M. AMUSSAT, *before a Commission of the Royal Academy of Medicine of Paris, consisting of* MM. Velpeau, Gerdy, Blandin, Barthélemy, Adelon, Moreau, and Bouillaud. *Reported by* M. BOUILLAUD.

[THE following are the experiments referred to in Article XII. of the present Number, page 446. Although we give an abstract of each experiment narrated, we have adopted a different arrangement from that employed by the reporter of the Commission.]

§ I. AIR ARTIFICIALLY INTRODUCED OR INJECTED.

A. *Air injected by means of a Syringe.* (Experiments 20, 23, 21, 22 of the Commission.) The animals not purposely debilitated before the experiment.

*Experiment* 1. Young bull-dog, of the average height. Laryngotomy performed previous to the experiment. Opening made into the right jugular. Venous pulse ascertained to exist. Having introduced into the wound of the vein a tube sufficiently large to prevent the exit either of air or blood, and provided two ligatures, by which the vein might be secured both above and below the wound, air was injected cautiously and gradually, by means of a moderate-sized syringe. When two-thirds only of the air had been injected, the animal was strongly agitated; the respiration became anxious and embarrassed, and the circulation accelerated; and, when all the air had been injected, a sound was heard in the heart, resembling a mixture of blowing and gurgling, ("un mélange de souffle et de glou-glou.") This sound disappeared in a minute; the respiration returned to its natural state; and, on the ligatures being applied, the animal walked quietly to its kennel. It still lives.

*Exp.* 2. Dog of ordinary size, slightly enfeebled by an experiment made ten days before. Left jugular vein opened in the middle of the neck, and a *small syringeful* of air injected into the cerebral end of the vein. Posture vertical. The dog struggled and howled: at the end of a minute he fell, and died.—*Inspection:* Bubbles of air in the mammary veins. Heart enormously distended; frothy blood in the right auricle, and in larger quantity in the right ventricle. Not a bubble of air in the left cavities of the heart, which contain a bright red blood. Venous blood in the inferior vena cava. Bubbles of air in the left jugular and facial veins, as also in the latter vein of the opposite side; (the right jugular had been obliterated in a former experiment.) Bubbles of air in the subclavian veins of both sides; also in the longitudinal sinus, and the veins which empty themselves into it, and in the superior petrosal sinuses.

*Exp.* 3. Dog of ordinary size. Injection, in half a minute, of somewhat more than fifteen cubic inches of air into the right jugular vein. The dog remains calm during the injection, and, on compressing the chest, a few bubbles of air pass out of the wound. Seventeen minutes after the first opening, the vein being again opened transversely, the air introduced itself with *lapping* sound. The wound is kept open and cleared of clots, and the air continues to enter. At the end of ten minutes from the second opening, the animal is set at liberty, and reserved for a second experiment ten days afterwards.

*Exp.* 4. Large mastiff. Opening made into the jugular vein at the middle of the neck: but little blood is lost, and no air enters. After two minutes, a small syringeful of air is introduced, without causing any agitation. A second syringe and a half full of air being introduced, the animal struggles violently, but shows no other symptom of the introduction of air. Another syringeful of air is now injected into the carotid artery. Convulsive movements and tetanic spasms, lasting some seconds, are followed by complete loss of power. Twenty-five minutes from the first opening, the animal is placed in the vertical position, and the jugular vein opened at its lower part. *Lapping* sound follows; the respiration becomes difficult; the animal falls, and discharges its fæces. In six minutes more, the respiration is convulsive and difficult; and after a few minutes the whole body is convulsed. One hour and forty-seven minutes from the commencement of the experiment, the animal was killed by opening the aorta.—*Inspection:* Serum in the pericardium; parietes of the heart flabby. Pure blood, without air, in the right cavities of the heart. Large air-bubbles in the superior vena cava. Some air-bubbles also in the veins of the brain and in the mammary veins.

[These last two experiments are mixed, and therefore inconclusive. In both the air was first injected, and then allowed to enter spontaneously: in the last the air was injected not only into the jugular vein, but also into the carotid artery.]

B. *Air injected by insufflation through a Tube.* (Experiments 24, 25, 26, 38, 39, 40 of the Commission.) The animals (dogs, horses, and one mule,) not purposely debilitated.

*Exp.* 1. Dog of moderate size. Air blown through a tube into the thoracic end of the right jugular vein. At conclusion of experiment, cries, struggles, panting, discharge of urine. Death in two minutes.—*Inspection:* Heart extremely distended; its right cavities filled with frothy blood; its left cavities quite free from it.

*Exp.* 2. Young dog of small size. Air blown gently into the thoracic end of the right jugular vein. Directly after the injection of air, cries, slight struggles, discharge of urine. Death at the end of one minute.—*Inspection:* Heart greatly distended; frothy blood in the right cavities; left cavities containing common blood.

*Exp.* 3. Small dog. Air blown gently into the thoracic end of the jugular vein. Immediately after, cries, violent struggles, and discharge of urine. Injection repeated at the end of three minutes, and followed by cries, falling of the head, and convulsive respiration. Death in half a minute from the second injection, and in three and a half minutes from the first.—*Inspection:* Strong contractions of the right auricle, which is distended, as well as the right ventricle. From the latter escape air and frothy blood. Frothy blood in the left cavities of the heart and in the aorta.

From these three experiments it appears that the insufflation of air from the chest of a man causes almost instantaneous death. In one instance, death ensued in one minute; in another, in two minutes; and in the third experiment, in three and a half minutes from the first introduction of air. The first experiment of the series (A, § I.) is the only one which admits of comparison with the three just detailed. In that experiment little inconvenience followed the injection from a syringe of a moderate quantity of air. In all the experiments in which air was introduced from the mouth, death was the prompt result. It is *probable,* then, that air which has been breathed proves more speedily and certainly fatal than air which has not been breathed. A mixed experiment, (No. 21 of the Commission,) in which air was first injected by a syringe, and then allowed to find its way into the vein, and this without fatal consequences, strengthens this probability.

As regards the post-mortem appearances, it will be remarked that air and frothy blood were found only in the right cavities of the heart in the first two experiments of the present series; and in these death took place after an interval of one and two minutes respectively. In the third experiment, death took place at the end of three minutes and a half, and frothy blood was found in both cavities of the left side of the heart and in the commencement of the aorta.

*Exp.* 4. Old horse. Left jugular vein opened at the middle of the neck. On introducing the tube, some air having entered, insufflation was practised immediately. After the second insufflation, the animal falls, struggles violently, and makes fruitless attempts to rise; frequent respiration, groans, convulsions. Death in six and a half minutes from the commencement of the experiment.—*Inspection.* Heart enormously distended. Much froth in the right ventricle, on the surface of a large clot. Frothy blood in the coronary artery and aorta. Blood, with very little air, in the left cavities. The entire venous system, not excepting the portal veins, containing a large quantity of air.

*Exp.* 5. Mare. Air twice breathed through a tube into the jugular. At the end of the second insufflation, the animal falls as if struck by a club, neighs, and expires between five and six minutes from the beginning of the experiment.—*Inspection.* Distension of the right cavities of the heart. Mixture of pure and frothy blood in these cavities; bloody froth in the pulmonary artery; a fine red froth in the left ventricle. Bubbles of air in the coronary veins; mixture of blood and froth in the aorta. Numerous bubbles of air in the veins of the convexity of the brain, and infiltration of air in the sub-arachnoid cellular tissue.

*Exp.* 6. Mule. Air breathed forcibly into the thoracic end of the divided jugular. Two insufflations; after the second of which the animal falls, rises again, totters, and again falls; struggles, and grows rigid. Four minutes from the commencement of the experiment, groans and convulsive respiration. Death in five minutes. —*Inspection.* Extreme distension of right cavities of the heart; frothy blood in all its cavities. Bubbles of air in the veins of the convex surface of the cerebrum, in those of the corpus callosum, and under the arachnoid.

The experiments just reported agree with similar experiments made on dogs. In dogs, death took place in from one to three and a half minutes; in horses, from five to six and a half minutes. The existence of air in almost all parts of the venous system, and more especially in the veins of the cerebrum in the horse, forms the chief difference between the two sets of experiments referred to.

### § II. AIR SPONTANEOUSLY INTRODUCED.

#### A. POSITION HORIZONTAL.

(*a.*) *Animals not debilitated before the experiment.* Experiments 1, 2, 3, 4, 5, (dogs,) 30, 31, 32, 33, (horses,) of the Commission.

*Exp.* 1. Dog, the size of a fox. Oblique incision made into the upper part of the right jugular vein. Venous pulse in the lower third of the vein, but not at the point of incision. No introduction of air. A puncture being now made in that part of the vein where the venous pulse existed, about one inch from the subclavicular vein,—introduction of air, with a sound resembling the *lapping* of a dog or cat. This sound exists chiefly during inspiration, but at certain moments corresponds with the *movements* of the heart. After ten minutes, the animal still living, the chest was opened, when the right auricle and ventricle were very greatly distended, but still contracting. After losing about three ounces of blood the animal expired, about twenty-seven minutes after the first incision. Death took place gradually, without loss of arterial blood, five or six minutes after opening the chest and pericardium.—*Inspection.* Frothy blood, of the colour of the lees of wine, in the right auricle. Black blood, without air, in the left auricle; left ventricle empty. Frothy blood in the pulmonary artery and its branches. Lungs healthy, but containing little blood. Escape of air-bubbles from the subclavicular and jugular veins.

*Exp.* 2. The sounds of the heart having been ascertained to be precipitated, but normal, an opening was made into the right jugular vein. Immediately afterwards the animal sighed, and air entered the open vein with a *lapping* sound, and the escape of frothy black blood. On auscultation of the heart, a double *bruit de souffle,* distinct from that just described, was heard. After an interval of three minutes, the same *bruit humide,* or splashing *(gargouillement),* which existed at the orifice of the vein was heard in the heart. After the lapse of another five minutes, the introduction of air was synchronous with the inspiration, and its exit with the expiration; but, at the end of another minute and a half, the entrance and exit of the air are synchronous with the pulsations of the heart. Still later, the inspirations are said to be very feeble; a double *bruit de souffle*, resembling that which takes place in certain valvular affections, is heard, and continues nine minutes after the closure of the vein by the finger. This *bruit de souffle* ascertained to depend on the heart, and not on the respiratory movements. The pressure of the stethoscope causes reflux of the blood through the orifice of the vein. The same reflux takes place even when the respiration has become ventral. Quantity of blood lost, about six or eight ounces. The animal is left to itself thirty-eight minutes after the beginning of the experiment. After the lapse of forty-seven minutes, it rises, stretches itself on its belly, raises the head, makes a few steps, and falls. Death four and a half days after the experiment.—*Inspection* thirty-six hours after death. No sign of decomposition. Purulent effusion in the pleuræ, but especially in the right. Right cavities of the heart distended. A digital depression exists at the union of the two ventricles, especially towards the apex. Right auricle and ventricle entirely filled by a firm clot, partly black but chiefly amber coloured, which extends into the two venæ cavæ and into the pulmonary artery; left ventricle contains a soft black clot. The left auricle contains a somewhat firm reddish-grey clot, which extends into the pulmonary veins, without any trace of inflammation. Tricuspid valve slightly red and thickened; mitral valve healthy. No uncommon appearances in the aorta; no emphysema in the lungs. Some of the lobules of the middle lobe of the right lung inflamed, and a puriform mucus exudes from the bronchi. In the opinion of the Commission, the dog did not die from the introduction of air, but in consequence of an accidental inflammation.

*Exp.* 3. Moderate sized bitch. Sounds of the heart before the experiment, distinct and without souffle. Left jugular vein opened at the lower part, where the venous pulse exists. Air enters with a slight *lapping* sound: about a minute after, struggles; anxiety; high, frequent, and convulsive respiration. Three minutes and ten seconds after, *lapping* sound more distinct than at first; slight *bruit humide*. After five minutes, the orifice of the vein being closed with the finger, apparent death, with persistence of the movements of the heart. The finger being removed, the respiration is gradually reestablished, the eyes brighten, and the animal revives; the blood is then removed from the wound, and the orifice dilated. The same introduction of air and exit of frothy blood take place; the respiration becomes somewhat embarrassed, and the animal is in the same state as before the first application of the finger. After twelve minutes, the finger is replaced on the orifice of the vein: the respiration becomes feeble, a sudden convulsive extension of the limbs takes place, the eyes are distorted, and the respiration ceases; the animal drops the head convulsively, the urine and fæces are passed involuntarily, and neither respiration nor contraction of the heart can be heard. Death five or six minutes after the second application of the finger, and a quarter of an hour after the opening of the vein.—*Inspection.* Slight vermicular movement of the heart. Right cavities so distended as to appear at least thrice the size of the left; vena cava also distended; bubbles of air in the cardiac veins. Pressure on the heart causes reflux of frothy blood into the sub-clavicular vein. On opening the right auricle, an escape of frothy blood, of a somewhat ochry colour; the left ventricle filled with a perfect froth. Small quantity of blood, without air, in the left cavities of the heart. Slight emphysema, especially on the anterior edge of the lungs. Right auriculo-ventricular opening nearly twice the size of the left.

*Exp.* 4. Dog of moderate size. Opening made into the jugular at about an inch from the chest. Abundant hemorrhage ensued, with the introduction of some bubbles of air during inspiration. The fore-leg having been separated from the chest, the aperture became gaping, and air immediately entered the vein with a noise. The opening was then closed, and the respiration of the animal being slightly affected, he was left to himself. The animal survived, and after ten days was submitted to another experiment.

*Exp.* 5. Young spaniel bitch, of ordinary size. Opening made into the jugular vein at its lower part. Two minutes after, the *lapping* sound heard. After another minute, struggles, especially when placed in the vertical position. Respiration calm. Large bubbles of air enter and pass out by the wound during the respiratory movements. At the end of three minutes, respiration very anxious and frequent. The animal being now left to itself, and the vein no longer patent, no more air appears to enter, though some blood still flows from the wound. On examining the heart, a distant and prolonged *bruit de râpe* is heard. Auscultation, repeated at the end of three minutes, discovers the same sound. At eleven minutes from the beginning of the experiment, respiration returned to its normal state. After another eight minutes, the *bruit de râpe* less distinct. The animal still loses blood. Twenty-three minutes from the commencement of the experiment, the animal utters a cry, is seized with convulsions, discharges his fæces, and falls into a state of apparent death. The vein being now secured, and cold aspersions employed, the respiration is slightly reestablished. Death in half an hour from the beginning of the experiment.—*Inspection.* Enormous distension of right cavities of the heart, which contain a large quantity of frothy blood.

*Exp.* 6. Old horse. Left jugular opened in the middle of the neck; no air introduced. Another opening made lower down, about eight inches from the sternum: immediate introduction of air, but in small quantity. A third opening having been made at the lowest part of the neck, the air entered immediately, with a very loud sound. Six minutes after the first opening, large bubbles of air pass from the wound. Five minutes after the third opening, convulsive respiration, panting, suffering. Ten minutes after, respiration more and more frequent. Fifteen minutes after, defecation, moaning, anxious respiration. Nineteen minutes after, the animal stamps and stumbles. Twenty minutes after, the legs bend under him; he falls, and passes a white milky urine. Twenty-nine minutes after, the head drops, and the respiration becomes irregular and plaintive. Death in thirty-five minutes from the commencement.—*Inspection.* Heart distended. Coronary veins containing air; froth in the right auricle and ventricle; in the latter, black fluid blood also. Bubbles of air adhere to the walls of the left ventricle. Air-bubbles in the sinuses of the dura mater and in the veins of the convexity of the cerebrum. Clot, with froth on its surface, in the superior longitudinal sinus.

*Exp.* 7. Very small old horse. Left jugular vein opened at its lower part: immediate entrance of air with loud noise; accelerated breathing. The animal, being walked about, lets fall his head, trembles, trips, recovers himself, and falls again, three minutes after the opening of the vein. In this position, he pants, stretches himself out, neighs repeatedly, is seized with convulsions, his nostrils expand, his eyes open widely, and he dies fourteen minutes from the commencement of the experiment.—*Inspection.* Much froth in the right cavities of the heart, and a clot in the right ventricle. No air in the left cavities nor in the coronary veins. Bubbles of air in the veins of the thorax, the inferior vena cava, the femoral veins, those of the convexity of the brain, and in the superior longitudinal sinus. Lungs collapsed; no emphysema.

*Exp.* 8. A mare. An opening, eight lines in length, having been made in the inferior part of the right jugular vein, the sound indicative of the entrance of air into the vein is heard. After two minutes, the respiration embarrassed. After ten minutes, the animal falls on its knees, and makes vain efforts to rise; the head is supported against a tree, and the neck flexed. Respiration more and more frequent and difficult. Twelve minutes after, blood no longer flows from the upper extremity of the vein; the position of the neck also prevents the admission of air. For an hour and a half the animal is left to itself; the respiration is difficult, the body is covered by sweat, and he groans from time to time. A clot which stopped the opening of the vein having been removed, and the neck extended, air again enters. After a few minutes, fixed and haggard eye, very frequent convulsive respiration, tongue protruded from the mouth, extension of the head and limbs. Death one hour and forty-four minutes after the opening of the vein.—*Inspection.* Extreme distension of the right cavities of the heart with a mixture of liquid and frothy blood. No air in the left cavities. Bubbles of air in the veins of the convex surface of the brain. Lungs collapsed; no emphysema.

*Exp.* 9. Mare, fifteen years old. Right jugular vein opened at its lower part. Entrance of air with sounds of gurgling and *glou-glou.* Aperture widely open. After a minute, the animal sighs, the respiration becomes panting, and the upper lip is extended and contracted as in yawning. After fifteen minutes, the animal lies down, rises directly, and again lies down; the respiration becomes more panting, the limbs are extended, the head raised; he struggles, stiffens, yawns, and sighs. Death twenty-eight minutes after the opening of the vein.—*Inspection.* Distension of the right cavities of the heart. Some froth in right auricle; a considerable quantity in right ventricle, as also in the left ventricle. Bubbles of air in large numbers in the veins of the cerebrum and cerebellum, and infiltrated under the arachnoid. Lungs sound.

(*b.*) *Animals debilitated before the experiment.* Experiments 6, 7, 8, 9, 10, 34, 35, 36, 37, of the Commission: the first five on dogs, the last four on horses.

*Exp.* 1. Large dog, weakened by previous loss of blood. Right jugular opened at the lower part. The opening, which was from two to three lines in diameter, being kept extended, the lapping sound was repeatedly and very distinctly heard; the animal stiffens, and respires convulsively. Death at the end of four and a half minutes.—*Inspection.* On opening the pericardium, right cavities observed to be greatly distended; feeble contractions in the auricle, lasting thirty-eight minutes. A reddish froth, resembling lees of wine beat up with air, found in the right auricle and ventricle. There was also free air in both cavities, and a recent clot in the right ventricle. Left ventricle free from blood and air. Frothy blood in pulmonary artery, but not in the pulmonary veins. Bubbles of air in the right common iliac vein.

*Exp.* 2. Dog enfeebled by opening the left crural artery. Pulse, before the loss of blood, from eighty-four to eighty-eight; after the loss of blood, ninety-six to one hundred. The right subclavicular vein being exposed, it is seen to swell during expiration, and become empty during inspiration; but, the breathing becoming tranquil, a movement of flux and reflux is observed, synchronous with the pulsations of the heart. A small opening, a line in diameter, being made into the vein, no lapping sound is heard; but the breathing becomes quickened and embarrassed. Soon after the opening is made, however, some bubbles of air, mixed with blood, escape from the wound. After the lapse of two minutes, the leg being extended, and the wound held open, the *lapping* sound is heard, and the respiration becomes more embarrassed. The lapping sound is synchronous with the inspiration. Five minutes after the opening, the inspiration has become calmer, in spite of the continual introduction of air. After eight minutes, the leg is placed in its natural position, and the wound closed. Auscultation reveals a distinct *bruit de souffle,* accompanied by a *bruit humide.* The animal moans, extends the head, its limbs are convulsed, and its inspirations are panting. Death in twenty-five minutes.—*Inspection,* a quarter of an hour after death. Distension of right cavities of the heart. Air in the right jugular. No contractions of right auricle: on opening this cavity, air and frothy blood escape. Right ventricle contains still more frothy blood, mixed with clots. The left cavities contain an unusually large quantity of blood, partly in clots and free from air. No emphysema of the lungs.

*Exp.* 3. Dog of average size, weakened by loss of blood from the carotid artery. Opening made into the lower part of the jugular vein: air enters with *lapping* sound. After a minute, quickened breathing. After two minutes, cries, struggles, discharge of urine, dropping of the head, convulsive trembling, especially of the right side. At the end of seven minutes, the animal tries to rise, succeeds after the lapse of twelve minutes more, and walks about.

*Exp.* 4. Bitch above the average size, enfeebled by loss of blood. Axillary vein opened. No venous pulse, and no movement corresponding to the respiration observed. The orifice being put on the stretch, the *lapping* sound is heard, and frothy blood issues from the wound; at the same time the breathing is quickened and embarrassed. Sound of the heart imperceptible. Nine minutes after the opening of the vein, the *lapping* sound ceases, though the clots are cleared away with a sponge. Respiration still frequent, but less difficult. At the end of twelve minutes the animal is left to itself, and is to all appearance quite well. The dog is then killed by making two large openings into the thorax.—*Inspection.* Heart contracts by a sort of vermicular movement. Through the walls of that viscus a sort of whirlpool can be distinguished, caused by a mixture of small bubbles of gas with the blood. On opening the right cavities there escapes liquid blood, with a few extremely minute air-bubbles. Small quantity of frothy blood in right ventricle, and still more in the pulmonary artery. Nothing remarkable in other organs.

*Exp.* 5. Spaniel of an ordinary size. The crico-thyroidean membrane and the sub-hyoidean muscles divided previous to the experiment. Sounds of the heart accelerated, but without souffle. Animal debilitated by the abstraction of blood from the right crural vein. The brachial vein having been exposed and opened at the lower margin of the axilla, was found to be set in motion, either by the venous pulse or by the subjacent artery. No introduction of air. The jugular vein is now opened at the part where the venous pulse exists: immediately afterwards, *lapping* sound, and at the end of half a minute the sounds of the heart indicate the presence of air in its cavities. In another half minute, anxiety, struggles. Three minutes after the opening of the jugular, the vein is secured. No longer any *glou-glou* in the heart. The animal left to himself. Forty-eight hours afterwards he is found enfeebled, but can support himself, and walk without much difficulty. He is again left to himself, and killed on the eighth day after the experiment.—*Inspection.* Right auricle greatly distended: it contains some bubbles of air. Bubbles of air also found in the right ventricle. Blackish clots of blood in the left cavities of the heart. The posterior mediastinum distended with air at its upper part; as also the axilla. Bubbles of air in the internal mammary, inferior vena cava, renal, and crural veins; and in the aorta, left iliac, and crural arteries.

*Exp.* 6. Horse debilitated by abstraction of blood from the carotid artery. Right jugular opened at the lower part of the neck. The sound indicative of the introduction of air is immediately heard, the breathing becomes accelerated, and after two minutes the animal falls, struggles violently, trembles, raises the head slightly, and again lets it fall. Some minutes afterwards, groans, discharge of urine. Death nine minutes after the opening of the vein.—*Inspection.* On dividing the skin covering the chest, escape of a large quantity of frothy blood from the veins, and of red blood from the arteries. Much froth and liquid blood in the right cavities of the heart, a large quantity of blood without intermixture of air in the left cavities. A clot in the aorta, with bubbles of air here and there on its surface. Coronary arteries and veins contain bubbles of air. Air-bubbles also in the femoral vessels, and in the inferior vena cava. Air also found in the veins of the convexity of the brain and in one of the ventricles. Lungs collapsed and flattened, free from emphysema.

*Exp.* 7. Horse enfeebled by loss of blood from the carotid. Jugular vein opened at the lower part of the neck, and secured by a ligature above the wound. Immediate entrance of air with usual sound. At the end of two minutes respiration accelerated. Two minutes later the animal is led about. The respiration soon becomes more frequent, he trips, falls on his knees, rests a moment in that position, and then falls on his side. At the end of eleven minutes, the eyes are closed, the nostrils largely open, the respiration difficult, and the limbs convulsed. Death twelve or thirteen minutes after the opening of the vein.—*Inspection.* Great distension of the right cavities of the heart by clots and frothy blood. No air in the left cavities. Air-bubbles in the veins of the convex surface of the cerebrum and in the superior longitudinal sinus. Lungs collapsed, and free from emphysema.

*Exp.* 8. Horse, debilitated by the abstraction of from three to four pounds of blood from the carotid. Large opening into the lower extremity of the jugular, followed by *glou-glou* and *gargouillement.* At the end of two or three minutes, accelerated and embarrassed respiration, sighing, and discharge of urine. At the end of eight minutes, the animal stumbles, as if drunk. Half a minute later, he falls, sighs, groans, and struggles. A minute and a half later, the groans become more plaintive, and the neck and forelegs are extended convulsively. Death thirteen minutes after first introduction of air.—*Inspection.* Right cavities of the heart distended by a large quantity of frothy blood. Air-bubbles on the walls of the left cavities, and in great numbers in the coronary veins. Bloody froth, resembling whipped cream tinged of a rose colour, in the pulmonary artery. Bubbles of air in the aorta. The surface of the cerebrum, cerebellum, and medulla oblongata studded with innumerable bubbles of air; some of which are deposited beneath the arachnoid, others contained in the veins. Nothing remarkable in the lungs.

*Exp.* 9. Horse, debilitated by abstraction of about four pounds of blood from the jugular. That vein being then opened at its lower part, the air enters with a gurgling and pulsating sound. At the end of a minute, embarrassed respiration. Eight minutes after, the animal stumbles, falls on its fore-knees, and then on its side. At the end of another six minutes, he extends his limbs convulsively, and dies sixteen minutes from the opening of the vein.—*Inspection.* Right cavities of the heart distended by bloody froth; the same bloody froth in the pulmonary artery; frothy blood in the coronary arteries and veins. Froth, without blood, in the left cavities; liquid blood and air-bubbles in the aorta. Air-bubbles, also, in the veins of the cerebrum, and in moderate quantity under the arachnoid.

B. POSITION VERTICAL.

(*a.*) *Animals not debilitated before the experiment.* Experiments 11, 12, 13, 14 of the Commission.

*Exp.* 1. A dog, of moderate size, meager and ill. Opening made into the lower part of the jugular. *Lapping* sound, much more distinct during inspiration. The animal being now placed in the vertical position, utters cries, and struggles. A minute afterwards, respiratory movements suspended; the pulsations of the heart, however, can be felt, and the flux and reflux take place in the vein. The animal makes two or three inspirations and expirations, during which blood issues from the wound. Death three minutes from the opening of the vein.—*Inspection.* Right auricle and ventricle filled with frothy blood. No frothy blood in the inferior cava. Numerous bubbles of air in the left jugular, and in the other veins of the neck as far as the facial.

*Exp.* 2. Healthy spaniel, of ordinary size. The right jugular vein opened. Introduction of air, at first without *lapping* sound; which, however, takes place in half a minute. The wound being filled with blood, the sound ceases. The animal is now placed in a vertical position: at the end of nine minutes, respiration frequent and agitated. The animal being now replaced in the horizontal posture, a large quantity of frothy blood issues from the vein. The hemorrhage is now arrested. The heart's beat irregular and intermittent; respiration convulsive, at one time rapid, at another slow, and even suspended. The animal is again placed in the vertical position, seventeen minutes after the opening of the vein; the aperture is freed from clots, and the air again enters with a *lapping* sound, still more distinct than before. The animal now seems to suffer more; the respiration is become stertorous. Twenty-one minutes from the commencement of the experiment, the animal is left to itself: he walks about, and discharges his fæces. At the end of thirty-six minutes, the wound is closed; the animal having lost but a moderate quantity of blood. At the end of a few seconds he falls on his side, breathing anxiously. At the end of some minutes he rises, and is then left to himself. At the end of three days the dog is killed, by dividing the aorta and vena cava with a bistoury.—*Inspection.* Heart flabby, especially the right side: no air in its cavities. No air in the jugular, coronary, and mammary veins; nor in the sinuses and veins of the brain.

[N.B. The death and dissection of this animal are put down as a separate experiment, with a view, probably, of completing the number of forty experiments. We have thrown these two, No. 12 and 13 of the Commission, into one.]

*Exp.* 3. Large dog. Opening made into right jugular vein, and followed instantly by the introduction of air. The animal being immediately placed in the vertical position, utters cries, loses its senses, and discharges its urine. It is again placed in the horizontal posture, and the mouth of the vein closed: the animal now remains motionless, with its eyes closed and its respiration suspended, having all the signs of apparent death. The ligature is now removed, and a large quantity of blood flows from the wound. After half a minute the respiration returns, at first convulsive, then calm, and the animal recovers. Seven minutes from the beginning of the experiment, the dog being thus recovered, is again placed in a vertical position. At the end of a minute the air again enters with a *lapping* sound: the respiration becomes very difficult, the animal moans, is in a state of complete exhaustion, and discharges its urine and fæces. In four minutes more, no sign of life. The animal being again placed in the horizontal posture, frothy blood issues from the vein, and the breathing is reestablished; with convulsions, first of the diaphragm, then of the whole body, the heart beats slowly but regularly. Death, sixteen minutes from beginning of experiment.—*Inspection.* Frothy blood, in large quantity, in the right cavities of the heart. Small quantity of red blood, without air, in the left cavities. Air in the vein of the cerebrum, and in the superior longitudinal and lateral sinuses. No air in the left jugular.

*Exp.* 4. Dog, thin and ill. After having applied a ligature to the right jugular vein, an opening is made into it near the thorax: air immediately enters with a *lapping* sound, and the breathing is accelerated. During expiration, large bubbles of air pass out from the aperture. The animal is now placed in a vertical position: the air continues to enter, and the respiration becomes quicker and quicker. Ten minutes after the opening made into the vein, cries, struggles, discharge of fæces. On examining the heart, *bruit de souffle.* After twenty-one minutes, the opening of the vein being closed, the respiration becomes more and more irregular and convulsive. Death, twenty-four and twenty-five minutes from commencement of experiment.—*Inspection.* Right cavities of the heart greatly distended by blood, in great part liquid, and less frothy than in other experiments. Origin of pulmonary artery filled with frothy blood. No air in the left cavities.

(*b.*) *Animal debilitated before the experiment.* (Exp. 16 of the Commission.)

*Exp.* Dog enfeebled by a previous experiment. Position horizontal. Large opening made into the lower part of the jugular vein, followed immediately by *bruit de lapement;* no struggles. At the end of half a minute, the animal, being placed in a vertical position, becomes strongly agitated, breathes with difficulty, and discharges its urine. After the lapse of two minutes more, the dog grows stiff, faints, lets fall the head, and again discharges his urine. He is again placed in the horizontal posture, and raised at the end of half a minute. The eye is now fixed and motionless; scarcely any sign of life exists; tetanic spasms, dropping of the head. Death at the end of about six minutes.—*Inspection.* Right cavities of the heart distended. Right auricle contains a clot of blood, filled with air-bubbles. A considerable quantity of frothy blood in the right ventricle. No air in the mammary veins.

### § III. Experiments showing the modifying and remedial Effects of Compression of the Cavities, and of Suction, on the Introduction of Air into the Veins.

*Exp.* 1. Small dog. The jugular being exposed, and the thorax compressed, the vein is found to swell at each compression. During a deep inspiration the vein becomes empty and flattened, and loses the colour due to the blood it contained. The jugular vein is now opened, but without effect; on enlarging the opening, however, a *lapping* sound is heard, and the animal struggles and becomes anxious. During a deep inspiration the *lapping* sound is heard more distinctly, and air-bubbles escape by the opening of the vein. Three minutes after making the opening into the vein the aperture is closed, and the heart exposed. When the heart is untouched, nothing escapes by the opening of the vein, but on compressing the thorax blood, mixed with air-bubbles, escapes from the wound. Death in ten minutes.—*Inspection.* The heart and vessels present the usual appearances, and frothy blood escapes from the right cavities of the heart.

*Exp.* 2. Shepherd's dog. The abdomen and chest are tightly bandaged, and the bandage secured by a tape; the right jugular vein is then opened in the situation of the venous pulse: a distinct *lapping* sound immediately follows, and after some seconds the respiration becomes difficult. At the end of two minutes the animal is placed in the vertical position, and the *lapping* sound becomes more distinct, and more frequent. The respiration is accelerated; anxiety, cries, and groans follow, and the head drops. The animal being now placed on the back, frothy blood issues from the wound. After six minutes the bandage is removed, and the *lapping* sound continues. The animal is now left to itself, but does not rise; being placed on the ground he stands up, walks, and again falls. After fifteen minutes, cries, convulsions, howlings, evacuation of the urine and fæces, dropping of the head, tongue hanging from the mouth. Blood, mixed with some air-bubbles, escapes during all this time. The quantity of blood lost is so considerable as to lead some of the assistants to attribute the death, which took place seventeen minutes from the commencement of the experiment, to this cause.—*Inspection* immediately after death. Distension of the right cavities of the heart with frothy blood. Some air-bubbles in the left cavities, air in the mammary, crural, venæ cavæ, and pulmonary veins, and in the mammary and pulmonary arteries.

*Exp.* 3. Spaniel bitch above the ordinary size. Opening made into the right jugular, and venous pulse observed to exist. After fifteen seconds, introduction of air, with *lapping* sound. The chest is now strongly and suddenly compressed, and a stream of blood, with some bubbles of air, issues from the lower end of the vein. The colour of the blood is brighter than that of venous blood. Two or three more compressions of the chest cause the escape of blood in less quantity and without appreciable mixture with air. After each compression the vein is secured to prevent a fresh introduction of air. The animal did not seem incommoded by the admission of air. The external wound being closed, the animal is left to itself. Two days afterwards, the animal walks about but seems in ill health. Death on the sixth day.—*Inspection* on the day of the death. Large quantity of free air in the right auricle. Clots, and black liquid blood, with numerous air-bubbles, but no froth, in the right ventricle. Numerous air-bubbles in the left ventricle, with a black, half-coagulated blood. No emphysema in the mediastinum. Fluid blood with numerous air-bubbles in the inferior vena cava. Large quantity of air and fluid blood in the crural arteries and veins.

*Exp.* 4. Dog of middle size. Incision made into the right jugular vein at the junction of the middle and lower thirds. Blood issues from the lower end during expiration, but no air enters. A tube of gum elastic is now introduced, as far as the point where the phenomena of flux and reflux are observed. Air now enters, and the respiration becomes difficult. At the end of ten minutes a longer tube is introduced as far as the auricle, and an attempt is made to withdraw the air by means of a syringe. A large quantity of blood, not frothy, but containing about a fifth of its volume of air is withdrawn. The animal has lost a good deal of blood. He is left to himself at the end of half an hour, and lies down with his legs extended, and is unable to move. Convulsions, difficult respiration, death.—*Inspection.* Distension of the right cavities of the heart by blood which is not sensibly frothy. Some frothy blood in the pulmonary artery. Blood without air-bubbles in the left cavities.

*Exp.* 5. Dog of ordinary size. Opening made into the lower part of the right jugular vein. A considerable quantity of blood escapes from the vein and the dog struggles violently. As the air does not enter, a new opening is made into a part of the vein nearer the chest, and the dog is placed in the vertical position. The opening is enlarged but no *lapping* sound is heard. Compression of the chest causes the escape of frothy blood. A little later, the *lapping* sound is heard, but the air evidently finds its way into the vein in smaller quantity than in the previous experiments. A tube of gum elastic is now introduced into the vein as far as the auricle, and blood mixed with foam is removed by means of a syringe. The tube is now removed and the vein secured. The animal which had had some alvine discharges is placed on the ground and walks unsteadily. The dog recovers, and is set at liberty.

*Exp.* 6. Large dog. Laryngotomy performed. Right jugular vein opened below its union with the facial, and about two inches above the position of the venous pulse. No introduction of air, a tube being now introduced into the vein air enters in small quantity during inspiration, and passes out during expiration. The same vein is now opened at its lower part, and the orifice kept gaping; the air enters slowly and silently at first, and then with distinct *lapping* sound. Struggles and accelerated breathing, then again calm respiration. The dog being now held upright and the head drawn back, struggles violently. Twenty-nine minutes after the last opening, a tube is introduced as far as the right auricle, and suction employed by means of a syringe. Pure blood only is extracted. (The syringe is out of repair.) In five minutes more respiration difficult. The dog is now left to itself, the vein being secured. He supports himself a moment, then falls on his side, and has tetanic convulsions of the limbs. Apparent death. A large quantity of water is now thrown over the animal, and he recovers, but still loses blood. Some time after, the respiration is stertorous, the dog stands up, makes a few steps, and then falls again. Convulsive movements with cessation of breathing follow, and apparent death. The animal is left for dead, but recovers on the following day and escapes.

[To these experiments of the commission we will add two experiments made by Nysten, bearing on the same points.]

*Exp.* 7. Large dog. Thirty-two cubic inches of air injected into the jugular vein. After some seconds, pulselessness, deep sighing, and apparent death. The subclavicular vein being now opened, and the chest compressed, a large quantity of air escapes. The animal immediately breathes, the pulse becomes perceptible, and he completely recovers. At the end of three days the dog is killed and no air is found in any part of the vascular system.

*Exp.* 8. Small bitch. Twenty cubic inches of air injected into the jugular. At the same moment, sound indicative of the mixture of air and blood in the right ventricle. After some seconds, cessation of breathing and pulse, and apparent death. A large incision is now made into the interior part of the thorax with a view of inspecting the body, but without any intention of restoring life. The subclavicular vein, as well as many smaller branches being opened, a large quantity of blood mixed with a small portion of air escapes, and the respiration and circulation are reestablished. The animal recovered from the immediate effects, but suffered much from the extensive wound. The third day he was killed, and the heart and large vessels were found free from air, and the lungs healthy. The experiments were frequently repeated with the same result.

*Bulletin de l'Academie Royale de Médecine.* 30 *Nov. et* 15 *Dec.* 1837.

---

### *On the Influence of Galvanism on the Process of Artificial Digestion.*
By Drs. PURKINJE and PAPPENHEIM, of Breslau.

ON examining with the microscope the products of the artificial digestion of muscular and nervous fibre, Drs. Purkinje and Pappenheim detected a large number of small oblong knotty bodies bearing a considerable resemblance to the seeds of some species of confervæ, and which they were at first inclined to consider as the product of the process, the commencement of a new organization. But on attentive examination of the mucous membrane of the abomasum, or fourth stomach, these minute bodies were found to constitute the parenchyma of innumerable glands, composing almost the entire substance of the mucous membrane. This structure becomes apparent by submitting shreds of the membrane to the action of a concentrated solution of carbonate of potash, by which its tissue becomes hardened. It may then be easily cut into thin segments, which, when inspected with the microscope, display this peculiar structure of the membrane, showing it to be almost wholly composed of a congeries of minute glands. When a portion of the membrane is associated with the requisite quantity of water and muriatic acid to constitute the digestive liquor, the digestive activity of the mixture is in the first place directed against the cellular tissue which connects these innumerable glandules, and which are thus disconnected so as to float freely in the liquor. Our experimenters having convinced themselves that the digestive principle is intimately connected with this mucous glandular membrane, instituted the following experiments with a view to determine whether it alone was sufficient for the process of artificial digestion:

The glandular membrane of the stomach was carefully separated from the subjacent tissues, and portions of it, varying in weight, were put into the same determinate quantity of water along with pieces of coagulated albumen. Artificial digestion did not take place: on the contrary, symptoms of decomposition speedily ensued. It is thus proved that the mucous membrane alone is not capable of producing artificial digestion; but, as this process ensues when a small quantity of muriatic acid is added, it becomes an object to ascertain by what organic process this acid is secreted in the living stomach. Microtomical investigation of this organ discloses no peculiar structure to which the secretion of the acid can be ascribed; and the natural inference is, that the congeries of glandules composing the mucous membrane secretes at once both gastric juice and acid. It occurred to Drs. Purkinje and Pappenheim that possibly the nerves of the stomach, by an influence similar to that of galvanism, might generate the acid, either from the articles of food, or from the saliva and mucus, or from the serum of the blood contained in the vessels of the mucous coat of the stomach; or, lastly, from the runnet or glandular mucous membrane itself. This question was investigated experimentally.

1. Two glasses, containing each two drachms of saliva, connected together by a moist cotton thread, were submitted to the action of a galvanic battery, consisting of thirty pairs of four-inch plates, the wire from the positive pole being introduced into one glass, and that from the negative pole into the other. The odour of chlorine soon became perceptible at the positive pole; and in twenty-four hours the acid reaction was very decided, and was owing to muriatic acid. The nature of the reaction at the negative pole is not mentioned, as it does not bear upon the subject under investigation. Into the acid portion of the saliva, three grains of runnet and three cubes of coagulated albumen were introduced, and the mixture was set aside in a temperature of 98° F. No change was observable in the albumen in the time usually required for artificial digestion; but in eight hours its edges had assumed the characteristic translucency of albumen about to be dissolved; in twenty-two hours the translucency had increased, and the substance of the albumen was as soft as gelatine, but there was no rounding off of the edges and corners. It thus appears, if there be any dynamic process in the stomach having an influence similar to that of galvanism, that part at least of the muriatic acid requisite for digestion may be furnished by the saliva.

2. Into each of two glasses, connected together by a moist cotton thread, one drachm of albumen and three drachms of distilled water were introduced. The mixture was then submitted to the galvanic action. The albumen collected round the wire of the positive pole in large coagulated flakes, and the liquid in its vicinity showed no acid reaction dependent on muriatic acid. In the negative glass there was no appearance of flakes: the liquid, on the contrary, appeared less viscid than at the commencement of the experiment, and showed a slight alkaline reaction, which in twenty-four hours became very powerful; whilst the acid reaction continued weak, probably, as was conjectured, because the acid was expended in coagulating the albumen.

3. Four drachms of nasal mucus were collected and washed with distilled water, and were then rubbed into an emulsion with an equal quantity of distilled water. The mixture was then divided and exposed in equal quantities, in two glasses, to the galvanic action. The result was the same as in the experiment with albumen. Masses of mucus collected round the positive pole, and the wire had occasionally to be freed from the adhering flakes, that the rest of the liquid might be exposed to the influence of the galvanism. In twenty-four hours the liquid had acquired a tolerably strong acid reaction, dependent on muriatic acid, and the contents of the negative glass appeared less viscid, and showed an alkaline reaction.

4. It is generally supposed (we shall not stop to enquire how far the supposition is correct) that on many organic surfaces, as on the inner surface of the uterus, on the serous and mucous membranes, the serum of the blood is separated directly from the blood contained in the vessels. It thus became a question whether the dynamic effect of the nerves might not be sufficient to separate an acid from the serum of the blood on the surface of the stomach; and in this way to create an agent capable of effecting digestion. A quantity of the serum of human blood was accordingly introduced into two glasses, and exposed to the galvanic influence. In twenty-four hours the serum at the positive pole showed a decided acid reaction, which was proved to be owing to muriatic acid. Into this acid liquor, three grains of runnet and three cubes of albumen were introduced, and the mixture was kept at the temperature of 98° F. The runnet was dissolved, and the albumen became soft and greasy, but it did not undergo solution. In order to ascertain whether the presence of serum operates as an obstacle to artificial digestion, four and a half grains of runnet were introduced into a glass containing two drachms of distilled water, along with three drops of muriatic acid, and a like quantity of albumen as in the preceding experiment. Two drachms of serum were then added: the changes undergone by the albumen were the same as in the preceding experiment; and it thus appeared that the presence of serum impedes artificial digestion, as without it the solution of the albumen would have been complete.

5. It still remained to determine whether, through the dynamic influence of the nerves, the acid necessary for artificial digestion might not be prepared from materials provided by the peculiar organic structure of the runnet itself. Three grains of dried pulverized runnet were mixed with two drachms of distilled water and exposed in two glasses to the galvanic action. The contents of one glass showed an acid, those of the other an alkaline reaction. Into each a piece of albumen was introduced; that in the acid liquid presented translucent edges in about sixteen hours, and in two hours more it was completely dissolved. No apparent change had taken place in the other. Thus it appears that runnet with the assistance of galvanism is capable of yielding acid sufficient to produce artificial digestion. A quantity of digestive liquor was prepared in the same way by galvanism, and divided into two parts. The one was replaced in communication with the galvanic battery, the other was set aside in the temperature of 98° F. Albumen was introduced into each, and in both artificial digestion ensued, but in the former more rapidly than in the latter. The solution of the problem whether the more rapid digestion in the former case was owing to the concurrent influence of the galvanism or to a greater secretion of acid, is reserved till a future opportunity. The enquiry whether the acid obtained from the runnet is the product of chlorides occurring in its composition, or whether the runnet freed from all chlorides is still capable of yielding an acid is likewise deferred.

From the above experiments it may be concluded:

1. That the assumption of a peculiar organ for the secretion of muriatic acid in the stomach is unnecessary.

2. That the secretions which are mixed with the food, namely, the saliva and mucus, and possibly also the serous exudation from the blood-vessels of the mucous coat of the stomach, yield chloride of sodium in quantities sufficient for the digestion of coagulated albumen.

3. That if the nervous action in the stomach is either identical with, or analogous to galvanism, it would be sufficient to account for the secretion of the quantity of muriatic acid requisite for digestion, without the assumption of a special organ of secretion.

In examining the influence of various mechanical agents in furthering artificial digestion it was found that comminution of the coagulated albumen, agitation of the mixture, or an increased pressure, in imitation of that produced upon the contents of the stomach by the muscles of the abdominal parietes, facilitated digestion The increased pressure was effected by the weight of the fluid in a barometer tube.

*Müller's Archiv.* 1838. Heft i.

